# Genome analysis for the identification of genes involved in phenanthrene biodegradation pathway in *Stenotrophomonas indicatrix* CPHE1. Phenanthrene mineralization in soils assisted by integrated approaches

**DOI:** 10.3389/fbioe.2023.1158177

**Published:** 2023-05-04

**Authors:** Alba Lara-Moreno, Francisco Merchán, Esmeralda Morillo, Jessica Zampolli, Patrizia Di Gennaro, Jaime Villaverde

**Affiliations:** ^1^ Department of Agrochemistry, Environmental Microbiology and Soil Conservation, Institute of Natural Resources and Agrobiology of Seville, Spanish National Research Council (IRNAS-CSIC), Seville, Spain; ^2^ Department of Microbiology and Parasitology, Faculty of Pharmacy, University of Seville, Seville, Spain; ^3^ Department of Biotechnology and Biosciences, University of Milano-Bicocca, Milano, Italy

**Keywords:** phenanthrene, *Stenotrophomonas indicatrix* CPHE1, mineralization, phenanthrene biodegradation pathway, genes expression

## Abstract

Phenanthrene (PHE) is a highly toxic compound, widely present in soils. For this reason, it is essential to remove PHE from the environment. *Stenotrophomonas indicatrix* CPHE1 was isolated from an industrial soil contaminated by polycyclic aromatic hydrocarbons (PAHs) and was sequenced to identify the PHE degrading genes. Dioxygenase, monooxygenase, and dehydrogenase gene products annotated in *S. indicatrix* CPHE1 genome were clustered into different trees with reference proteins. Moreover, *S. indicatrix* CPHE1 whole-genome sequences were compared to genes of PAHs-degrading bacteria retrieved from databases and literature. On these basis, reverse transcriptase-polymerase chain reaction (RT-PCR) analysis pointed out that cysteine dioxygenase (*cysDO*), biphenyl-2,3-diol 1,2-dioxygenase (*bphC*), and aldolase hydratase (*phdG*) were expressed only in the presence of PHE. Therefore, different techniques have been designed to improve the PHE mineralization process in five PHE artificially contaminated soils (50 mg kg^−1^), including biostimulation, adding a nutrient solution (NS), bioaugmentation, inoculating *S. indicatrix* CPHE1 which was selected for its PHE-degrading genes, and the use of 2-hydroxypropyl-β-cyclodextrin (HPBCD) as a bioavailability enhancer. High percentages of PHE mineralization were achieved for the studied soils. Depending on the soil, different treatments resulted to be successful; in the case of a clay loam soil, the best strategy was the inoculation of *S. indicatrix* CPHE1 and NS (59.9% mineralized after 120 days). In sandy soils (CR and R soils) the highest percentage of mineralization was achieved in presence of HPBCD and NS (87.3% and 61.3%, respectively). However, the combination of CPHE1 strain, HPBCD, and NS showed to be the most efficient strategy for sandy and sandy loam soils (LL and ALC soils showed 35% and 74.6%, respectively). The results indicated a high degree of correlation between gene expression and the rates of mineralization.

## 1 Introduction

Phenanthrene (PHE) is a low molecular weight polycyclic aromatic hydrocarbon (PAH) constituted by three fused benzene rings. It is mainly produced from anthropogenic sources such as oil mining, accidental discharge, pipeline vandalism, and poor municipal waste management causing adverse effects on public health and the environment (Prabhu and Phale, 2003; Varjani and Upasani, 2017). PHE is characterized by its ubiquity in the environment, and it is a pollutant of concern due to its persistence and toxicity, causing detrimental biological and environmental effects (Abdel-Shafy and Mansour, 2016); indeed, it has been included in the hazardous substances lists of the United States Environmental Protection Agency and the European Union (EPA, 2022).

In the environment, PAHs are removed in different ways such as photo-oxidation, chemical oxidation, bioaccumulation, volatilization, or adsorption to soil particles (Parab and Phadke, 2020). However, biological strategy is the main approach to removing PAHs in terrestrial and aquatic systems (Churchill et al., 2008; Nwankwegu et al., 2022), since microbial degradation is a cost-effective and eco-friendly technology (Brown et al., 2017).

Several studies showed PHE biodegradation in soil fostered by the presence of different bacterial genera: *Bacillus* (Mandree et al., 2021), *Massilia* (Gu et al., 2021), *Sphingomonas* (Waigi et al., 2015), or *Rhodococcus* (Wang et al., 2021). Additionally, bacterial strains from *Stenotrophomonas* genus have been described as PHE degraders (Kumari et al., 2017; Elufisan et al., 2020), such as *S. maltophilia* (Gao et al., 2013; Zafra et al., 2014; Xiao et al., 2021). However, as far as we know, the study of Lara-Moreno et al. (2021) was the first which demonstrated the ability of *S. indicatrix* to degrade PHE in solution. *Stenotrophomonas indicatrix* is a Gram-negative species that can be isolated from sunflower roots (Adeleke et al., 2021), soils (Lara-Moreno et al., 2021; Lozano-Andrade et al., 2021), oligotrophic water ponds (Friedrich et al., 2021), or from food-associated sources (Weber et al., 2018).

The degradation of PAH by bacteria is usually catalyzed by oxygenase and dehydrogenase enzymes. For instance, these enzymes include naphthalene dioxygenase (*NahAc*) and naphthalene monooxygenase (*NdoB)* (*Pseudomonas putida* G7, Cébron et al., 2008); pyrene dioxygenase (*Mycobacterium* sp. strain PYR-1, Stingley et al., 2004); dihydrodiol naphthalene dehydrogenase (*NahB*) (*Pseudomonas stutzeri AN10,* Bosch et al., 1999) or naphthalene dehydrogenase (*NahB*) (*P. putida* AK5, Izmalkova et al., 2013). Nevertheless, little is known about the genes responsible for PHE degradation in *Stenotrophomonas* genus. As far as we know, only Kumari et al. (2017) and Elufisan et al. (2020) have conducted research about the genes involved in PAHs biodegradation pathway in *Stenotrophomonas* genus and there are no scientific studies on *Stenotrophomonas indicatrix* species.

In the environment, the natural ability of soil is often exploited to remove contaminants. However, since natural attenuation is a very slow biological process for the restoration of contaminated soils (Zabbey et al., 2017), (bio)remediation techniques can be applied, including biostimulation, application of availability enhancers, and bioaugmentation. Biostimulation requires the addition of nutrients or electron acceptors into the soil. This option optimizes the strategy and critical factors for the remediation of polluted sites, including restricted oxygen, nutrient transfer, and competition between microorganisms (Masy et al., 2016). Regarding availability enhancers, PHE is considered a pollutant highly persistent in soil due to its strongly hydrophobic character (Humel et al., 2020). Therefore, the high adsorption could cause a decline in the PHE bioavailable fraction, which means a slowdown in the bioremediation progress (Humel et al., 2017). To counter these negative influences, bioavailability enhancers such as cyclodextrins (CDs), can be applied to the soil (Madrid et al., 2019; Madrid et al., 2022). The use of CDs is a greener alternative to organic solvents or synthetic surfactants used by several authors to remediate contaminated soils by PAHs (Sun et al., 2014; Liu et al., 2018). CDs have the capacity to form inclusion complexes with hydrophobic contaminants enhancing their water solubility and improving their elimination from soils (Morillo et al., 2020).

Bioaugmentation is considered a promising tool to enhance pollutant biodegradation. Bioaugmentation consists of the addition of exogenous or endogenous pollutant degraders into the contaminated site to accelerate contaminant removal (Tyagi et al., 2011). It has been recommended as the most suitable alternative to be used in bioremediation (Nwankwegu et al., 2022), using bacterial strains isolated from large-scale polluted areas where endogenous microbiota is generally adapted (Mrozik and Piotrowska-Seget, 2010). For this reason, in the present study, a bacterial strain isolated in our lab from highly contaminated industrial soil was selected for its capacity to degrade and mineralize PHE in an aqueous solution (Lara-Moreno et al., 2021). In the previous paper, the isolated bacterial strain was classified as *Stenotrophomonas maltophilia* CPHE1 (NCBI number: MT138842), but later, a more in-depth genetic study revealed that its classification corresponded to *S. indicatrix* (Lara-Moreno et al., 2023). Weber et al. (2018) classified for the first time this species as *S. indicatrix*, although they had previously classified it also as *S. maltophilia* due to their high genomic similarities. Up to now, no work has shown PHE degradation and mineralization to CO_2_ and H_2_0 in the soil in the sole presence of *S. indicatrix*.

Therefore, this study aimed to characterize *S. indicatrix* CPHE1 at the genomic level to classify genes annotated as dioxygenases, monooxygenases, and dehydrogenases into phylogenetic trees to detect putative PHE-degrading genes. Moreover, *S. indicatrix* CPHE1 whole-genome sequences were compared to genes of PAHs-degrading bacteria retrieved from databases and literature. The expression of selected genes such as *bphC*, and *phdG* genes (encoding for an extradiol dioxygenase, and a hydratase-aldolase) was studied by RT-PCR for their involvement in the PHE degradation pathway. On this basis, several eco-friendly bioremediation treatments were conducted using biostimulation, bioaugmentation with *S. indicatrix,* and the use of CDs to enhance the extent and rate of PHE biodegradation and mineralization in five contaminated soils with different physicochemical properties.

## 2 Materials and methods

### 2.1 Materials

PHE (C_14_H_10_, purity 98%) was purchased from Sigma-Aldrich (Madrid, Spain) and radiolabeled compound ^14^C-PHE (36 mCi⋅mmol^−1^, purity 99.9%, and radiochemical purity 100%) was acquired from the Institute of Isotopes (Budapest, Hungary). HPBCD was obtained from CycloLab Cyclodextrin Research and Development Laboratory Ltd. (Budapest, Hungary).

Mineral Salt Medium (MSM) composition (g L^−1^): 4.0 Na_2_HPO_4_; 2.0 KH_2_PO_4_; 0.8 MgSO_4_; 0.8 NH_4_SO_4_. The micronutrients solution (SNs) was composed of (mg L^-1^): 12.5 NiCl_2_ 6H_2_O; 25.0 SnCl_2_ 2H_2_O; 12.5 ZnSO_4_ 7H_2_O; 12.5 Al_2_(SO_4_)_3_ 18H_2_O; 75.0 MnCl_2_ 4H_2_O; 12.5 CoCl_2_ 2H_2_O; 37.5 FeSO_4_ 7H_2_O; 10.0 CaSO_4_ 2H_2_O; 3.75 KBr; 3.75 KCl; 2.50 LiCl (Fenlon et al., 2011). The mixture of MSM and SNs (50:1) was named NS in the study.

Five soils (PLD, LL, ALC, CR, R) with diverse physicochemical properties were collected from different points in the South of Spain. PLD soil is an agricultural soil located in Los Palacios y Villafranca—Seville (37°10′20.0″N 5°55′21.9″W), where wheat, cereals, and vineyard crops are mainly cultivated. The agricultural soil LL from Vejer de la Frontera—Cádiz (36°17′52.6″N 5°52′45.2″W) is devoted to intensive agriculture of carrot, cotton, leek, etc. ALC soil was taken from Alcornocales Natural Park (36°20′54″N 5°36′14″O) and is characterized by high organic matter (OM) content. CR soil is an agricultural soil from an area of olives within the experimental farm La HAMPA (IRNAS-CSIC) (37°17′28.3″N, 6°3′55.4″W). R soil, collected from a palm trees zone in Conil de la Frontera—Cádiz (36°18′32.4″N, 6°08′58.6″W), has been managed with a huge number of insecticides. The soil samples collection was conducted from the superficial horizon (0–20 cm), air-dried for 24 h at room temperature, and sieved (2 mm). Table 1S shows the physicochemical properties of the studied soils and their textural classification. The pH was determined in a proportion of 1:2.5 soil/water extract. The particle size distribution was evaluated using Bouyoucos densimeter; the calcination or muffling method was used to estimate the OM content of the soil weight loss on ignition (LOI) or calcination, the quantification of OM was determined by K_2_Cr_2_O_7_ oxidation, and the manometric method was used to measure the total carbonate content.

The PHE-degrading microorganism selected, *S. indicatrix* CPHE1, corresponds to *S. maltophilia* CPHE1 (NCBI number: MT138842) published by Lara-Moreno et al. (2021). Subsequent studies indicated greater phylogenetic closeness with the species *S. indicatrix* rather than *S. maltophilia* (Lara-Moreno et al., 2023)*.* CPHE1 strain was isolated in our lab from a highly contaminated industrial soil as reported by Lara-Moreno et al. (2021). The sequencing data of S. indicatrix CPHE1 were deposited in the National Center for Biotechnology Information (NCBI), under the BioProject ID PRJNA868539. The whole genome shotgun generated has been deposited in DDBJ/ENA/GenBank under the accession number JANQDV000000000. The version described in this paper is version JANQDV010000000. The raw data was deposited in the Sequence Read Archive (SRA) under the accession number SRR21098193 (Lara-Moreno et al., 2023).

### 2.2 Methods

#### 2.2.1 Bioinformatic analysis: nucleotide and protein sequence analysis

The sequences annotated as monooxygenases, dioxygenases and dehydrogenases were individually aligned against PDB (Protein Data Bank) database (Berman et al., 2000) and BLASTp of NCBI pipeline to identify reference sequences. References proteins were selected based on percentage similarity.

Subsequently, the selected reference proteins together with CPHE1 proteins were aligned using Clustal Omega, a multiple sequence alignment program (Sievers et al., 2011). The used format was ClustalW with character counts (alignment format with base/residue numbering).

Monooxygenase, dioxygenase, and dehydrogenase phylogenetic trees were constructed using the maximum-likelihood method selected from the package MEGA (Molecular Evolutionary Genetic Analysis) version 7 (Kumar et al., 2016). The employed parameters were the following: JTT matrix, gamma distribution of mutation rates with gamma optimized to 2, and the tree robustness was assessed using 1000 bootstrap replicates.

#### 2.2.2 RNA extraction, DNase treatment, cDNA synthesis, and Real-Time PCR (RT-PCR) analysis from *S. indicatrix* CPHE1

To evaluate the induction of PHE degrading genes, CPHE1 cells were collected by centrifugation (12,000 rpm for 10 min) at different times (0, 1, 3, 7, 14, and 21 days) of growth, after its cultivation in MSM supplemented with 10 mg L^−1^ PHE. The supernatant was removed, and RNA was extracted from the bacterial pellet by High Pure RNA Isolation Kit (Roche Molecular System, Switzerland). RNA samples from different times were treated first with DNAse, RNase-free (ThermoFisher Scientific, Massachusetts, EE.UU.), using 3U of DNase for 3 μg of RNA. Then complementary DNA (cDNA) synthesis was conducted using RevertAid First Strand cDNA Synthesis kit (ThermoFisher Scientific, Massachusetts, EE.UU.). The total RNA and the random hexamer primers were denatured at 65 C for 5 min. The remaining reagents (Buffer 5x, 20 U μL^−1^ of RNase Out, 10 mM dNTPs, and 200 U μL^−1^ retrotranscriptase enzyme) were added and incubated at 25°C for 5 min, 42°C for 60 min and kept at 70°C for 5 min to inactivate the enzyme. cDNAs were amplified with specific primers (Supplementary Table S2) for different PCR cycles depending on the analyzed gene (11–13 for 16s rDNA, and 30 cycles for considered degrading genes). 16S rDNA was used as a constitutive control of gene expression in *S. indicatrix* CPHE1. All RT-PCR experiments were performed in triplicate.

Specific PCR primers (Supplementary Table S2) for degrading genes were designed *in silico* using the program Primer3 (primer3 https://primer3.ut.ee/). Designed primers were checked by primer-BLAST tool.

#### 2.2.3 Phenanthrene degrading-microbial characterization of original soils

The total viable PHE degrading-bacteria in the studied soils were enumerated using the unit of measure, colony forming units per Gram of soil (CFU g^−1^ soil). 1 g of soil was added to 5 mL of MSM, and then 100 µL of the suspension was serially diluted (1:10). Aliquot (100 µL) of the resultant solutions were spread over petri dishes with MSM agar supplemented with 50 mg L^−1^ of PHE and incubated at 30°C ± 1. The plate count was conducted after 7 days.

#### 2.2.4 Inoculum preparation of *S. indicatrix* CPHE1 for phenanthrene mineralization in soils

*Stenotrophomonas indicatrix* CPHE1 grown in LB agar plate was transferred to Luria−Bertani (LB) broth. The culture was incubated under orbital shaking (160 rpm) at 30°C. After 20 h (at the end of the exponential phase), CPHE1 strain was centrifuged and then washed twice using MSM to remove any LB residues. Then, the cell pellet was resuspended in the necessary volume of MSM to obtain a 10^8^ CFU mL^−1^ density.

#### 2.2.5 Phenanthrene mineralization test in soils

Mineralization studies using ^14^C-ring-labelled PHE were performed in the five studied soils (PLD, LL, ALC, CR, R) under slurry suspension condition. Tests were performed in triplicate, using respirometers, which consist in a modified 250 mL Erlenmeyers with a soda tramp, containing 1 mL of 0.5 N NaOH. All the microcosm components were sterilized (Matachana steam sterilizer model S100 with one cycle at 120°C, pressure of 101 kPa, for 20 min), except the investigated soil, to preserve their endogenous microbiota. 10 g of each soil were added to Erlenmeyer flasks. Soils were artificially contaminated with a mixture of ^14^C-PHE (450 Bq per flask) and unlabeled PHE to obtain a total concentration of 50 mg kg^−1^. 0.5 mL of a 1000 mg L^−1^ PHE stock solution in methanol, which also contained ^14^C-PHE (450 Bq), was added to 2.5 g of soil (25% of the total soil) and was stayed under a fume hood for 24 h at room temperature to evaporate the methanol. Subsequently, the remaining soil (7.5 g) was mixed, to avoid damage to soil endogenous microbiota. Finally, 50 mL MSM and 1 mL of SNs were added to Erlenmeyer flask and incubated at 30°C ± 1°C, under continuous slow stirring for 120 days.

Mineralization studies were conducted as follows: (A) Control: soil without treatment; (B) Biostimulation: soil + NS; (C) Bioaugmentation: soil + NS + *S. indicatrix* CPHE1 (10^8^ CFU g^−1^); (D) Bioavailability enhancer: soil + NS + HPBCD solution (10 times the initial molar concentration of PHE); (E) Mixing techniques of biostimulation, bioaugmentation and bioavailability enhancer: soil + NS + *S. indicatrix* CPHE1+ HPBCD. Simultaneously, abiotic controls were set adding 200 mg L^−1^ of HgCl_2_ to the soil, to monitor abiotic PHE dissipation.

Samples were periodically taken to monitor the formation of ^14^CO_2_ trapped in the alkali trap. NaOH solution from the trap was mixed with 3 mL of a liquid scintillation cocktail (Ready safe from PerkinElmer, Inc., United States). Samples were kept in darkness for 24 h to dissipate the chemiluminescence. A liquid scintillation counter (Beckman Instruments Inc., Fullerton, California, model L55000TD) was used to measure the radioactivity.

The mineralization curves were adjusted to three kinetic models: a simple first-order model (SFO), a biphasic first-order sequential model (Hockey-Stick, HS), and a first-order multi-compartment model (FOMC). These models were selected according to the FOCUS (2006) work group. The Solver tool (included in Microsoft Excel statistical software) and the equations described in Lara-Moreno et al. (2022a) were used, depending on the kinetic model that fits the best to each experimental set of data. Kinetics parameters were obtained from kinetic models: k_1_, k_2_, α, β (mineralization rate constants), tb (time at which rate constant changes), and DT_50_ (time taken for 50% of substance to disappear by dissipation processes). The chi-square (χ2) test was calculated as an indicator of the goodness of fit (χ2 values should be < 15 to mean a good fit).

#### 2.2.6 Phenanthrene availability in soil

NS and HPBCD solutions were used to determine their effect on soil PHE bioavailability. For this purpose, 1 g of soil contaminated with 50 mg kg^−1^ of PHE was added to corex glass centrifuge tubes. Samples were extracted with 5 mL of NS (MSM + SNs) or NS + HPBCD (10 times the molar concentration of PHE previously added in soil). The corex tubes were agitated on an orbital shaker for 72 h at 20°C ± 1°C and subsequently centrifuged (10 min, 7000 rpm) to separate the supernatant from the soil (Villaverde et al., 2012). The supernatant was filtered through a 0.22 μm Millipore glass fiber membrane. Then, 3 mL of the sample was taken and placed into a glass tube with 2 mL of hexane, which was shaken using a vortex device for 1 min and for 5 min in an ultrasonic bath. The hexane phase was placed in 2.5 mL glass vials. PHE concentration was measured using a gas chromatographer (Agilent GC 6890N) equipped with a mass spectrometer (MS, Agilent MD 5975B). The analytical method was described by Lara-Moreno et al. (2021).

## 3 Results and discussion

### 3.1 Bioinformatic analysis of genes involved in phenanthrene degradation pathways

Lara-Moreno et al. (2023) provided relevant information about the genome. The genome of *S. indicatrix* CPHE1 deposited in NCBI (JANQDV000000000) (Lara-Moreno et al., 2023) consists of 163 contigs, with a size of 4.553.664 bp, 66.1% of Guanine-Cytosine (G-C) content, and 4137 genes described.

Genes potentially associated with PHE biodegradation were bioinformatically predicted in *S. indicatrix* CPHE1 genome by two diverse strategies: (i) searching for specific annotation of gene products by RAST server and subsequently clustering them into phylogenetic trees using reference sequences; a bibliographic and database search of enzymes of other bacteria has been carried out to identify reference sequences. Data obtained allowed the improvement of the genome automatic annotation of the CPHE1 strain genes. Similarities between the whole set of selected enzymes have been analyzed by Clustal Omega obtaining a phylogenetic analysis. (ii) Analyzing the sequence homology with respect to reference sequences of bacterial strains able to biodegrade PHE (Cébron et al., 2008; Izmalkova et al., 2013) or bacteria frequently detected in the water systems by other studies (Stingley et al., 2004; Elufisan et al., 2020).

Considering the first approach, Supplementary Figure S1 shows the distribution of the annotated genes in different subsystems of *S. indicatrix* CPHE1 genome. Particular attention was focused on the subsystem of genes that participate in the aromatic metabolism compounds (boxed in red); these annotated genes could take part in the central catabolism of the metabolites formed during the degradation of aromatic compounds. Beta-ketoadipate pathway is an aromatic compound degradation route widely distributed in soil bacteria and fungi. On the one hand, catechol 1,2-dioxygenase converts catechol generated from aromatic hydrocarbons into intermediates of the tricarboxylic acid cycle (TAC). On the other hand, protocatechuic acid is turned into intermediates of the TAC catalyzed by protocatechuate 3,4-dioxygenase. Both enzymes act by cleaving aromatic ring. Catechol-1,2-dioxygenase, has been studied in species belonging to *Pseudomonas* genus due to its ability to cleave the aromatic ring of catechol molecule (Rodríguez-Salazar et al., 2020). Other annotated genes in the *S. indicatrix* CPHE1 genome could encode enzymes that participate in the salicylate and gentisate pathway, compounds that have been identified as intermediaries of PAH catabolism (Grund et al., 1992). The salicylic acid pathway has been described for several PHE-degrading bacterial genera or other PAHs, such as *Pseudomonas* (Lin et al., 2014), *Streptomyces* (Ishiyama et al., 2004), or *Stenotrophomonas* (Huang et al., 2013). Several authors have described monooxygenases (Medić et al., 2020) and dioxygenases (Moody et al., 2005; Gao et al., 2013; Kumari et al., 2017) as enzymes responsible for catalyzing the initial reaction of PAHs degradation pathway to produce hydrodiol or dihydrodiol.

**FIGURE 1:**
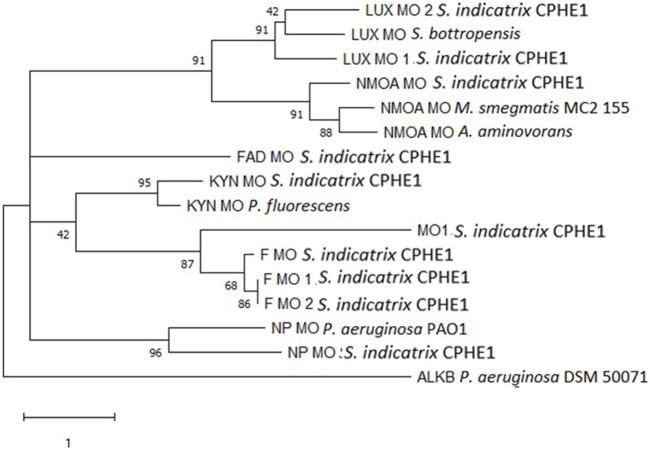
Maximum Likelihood tree based on the monooxygenase protein sequences annotated in the genome of *S. indicatrix* CPHE1 and on the reference monooxygenases involved in the degradation of polycyclic aromatic hydrocarbons. Bootstrap values (>30%) are expressed as percentages of 1000 replicates. Same branches representation was recovered by the neighbor-joining and maximum-parsimony algorithms. The scale bar indicates 1 substitution per nucleotide position in protein sequence tree. Phylogenetic tree was built using MEGA 6.

For this reason, we initially focused on the study of genes annotated as monooxygenases in the genome of *S. indicatrix* CPHE1. A phylogenetic analysis was carried out using the proteins reported in Table 1 (Figure 1). The phylogenetic tree demonstrated no similarities between the annotated CPHE1 sequences and the reference monooxygenase (AlkB*,* UniProtKB: A4XPE8), described in *Pseudomonas aeruginosa* P6 as responsible for petroleum hydrocarbon degradation (Wang et al., 2019; Medić et al., 2020). Since the other reference sequences were not involved in PAH degradation pathways, it has been ruled out that monooxygenases encode the first step in the PHE degradation.

**TABLE 1:** Monooxygenases annotated in *S. indicatrix* CPHE1 genome and reference monooxygenases (literature, pdb, NCBI, etc.).

Name	Function	Strain	References
MO1	Putative monooxygenase	*Stenotrophomonas indicatrix* CPHE1	-
F_MO	Flavin-dependent monooxygenase ArsO associated with arsenic resistance	*Stenotrophomonas indicatrix* CPHE1	-
F_MO1	Flavin-dependent monooxygenase ArsO associated with arsenic resistance	*Stenotrophomonas maltophilia*	Jensen et al. (2012)
F_MO2	Flavin-dependent monooxygenase ArsO associated with arsenic resistance	*Stenotrophomonas maltophilia*	Jensen et al. (2013)
FAD_MO	monooxygenase, FAD-binding	*Stenotrophomonas indicatrix* CPHE1	-
KYN_MO	Kynurenine 3-monooxygenase	*Stenotrophomonas indicatrix* CPHE1	-
KYN_MO	Kynurenine 3-monooxygenase	*Pseudomonas fluorescens* KMOs	Kim et al. (2018)
NMOA_MO	Nitrilotriacetate monooxygenase component A	*Stenotrophomonas indicatrix* CPHE1	-
NMOA_MO	Nitrilotriacetate monooxygenase component A	*Mycolicibacterium smegmatis* MC2 155	Zhang et al. (2011)
NMOA_MO	Nitrilotriacetate monooxygenase component A	*Aminobacter aminovorans* ATTCC 29600	Knobel et al. (1996)
LUX_MO	Luciferase-like monooxygenase YhbW	*Streptomyces bottropensis*	Maier et al. (2014)
LUX_MO_1	Luciferase-like monooxygenase YhbW	*Stenotrophomonas indicatrix* CPHE1	-
LUX_MO_2	Luciferase-like monooxygenase YhbW	*Stenotrophomonas indicatrix* CPHE1	-
NP_MO	2-nitropropane monooxygenase	*Stenotrophomonas indicatrix* CPHE1	-
NP_MO	2-nitropropane monooxygenase	*Pseudomonas aeruginosa* PA01	Salvi et al. (2014)
ALKB	Alkane-1-monooxygenase	*Pseudomonas aeruginosa* P6	Wang et al. (2019)

A similar approach was then undertaken for another enzyme class since the initial reaction of PAH biodegradation in *S. indicatrix* CPHE1 could be catalyzed by a multicomponent system of a dioxygenase, producing dihydrodiol (Kumari et al., 2017). This hypothesis is supported by two fundamental facts; first, the presence of similar dioxygenases has been detected in several PAHs degrading bacteria such as *Pseudomonas* (Prabhu and Phale, 2003; Cébron et al., 2008), *Mycobacterium* (Stingley et al., 2004; Moody et al., 2005), and *Stenotrophomonas* (Kumari et al., 2017). Second, the metabolite dihydroxyphenanthrene was identified during the PHE biodegradation of *S. indicatrix* CPHE1 (Lara-Moreno et al., 2021). These data would suggest the intervention of dioxygenase in PHE biodegradation.

**FIGURE 2:**
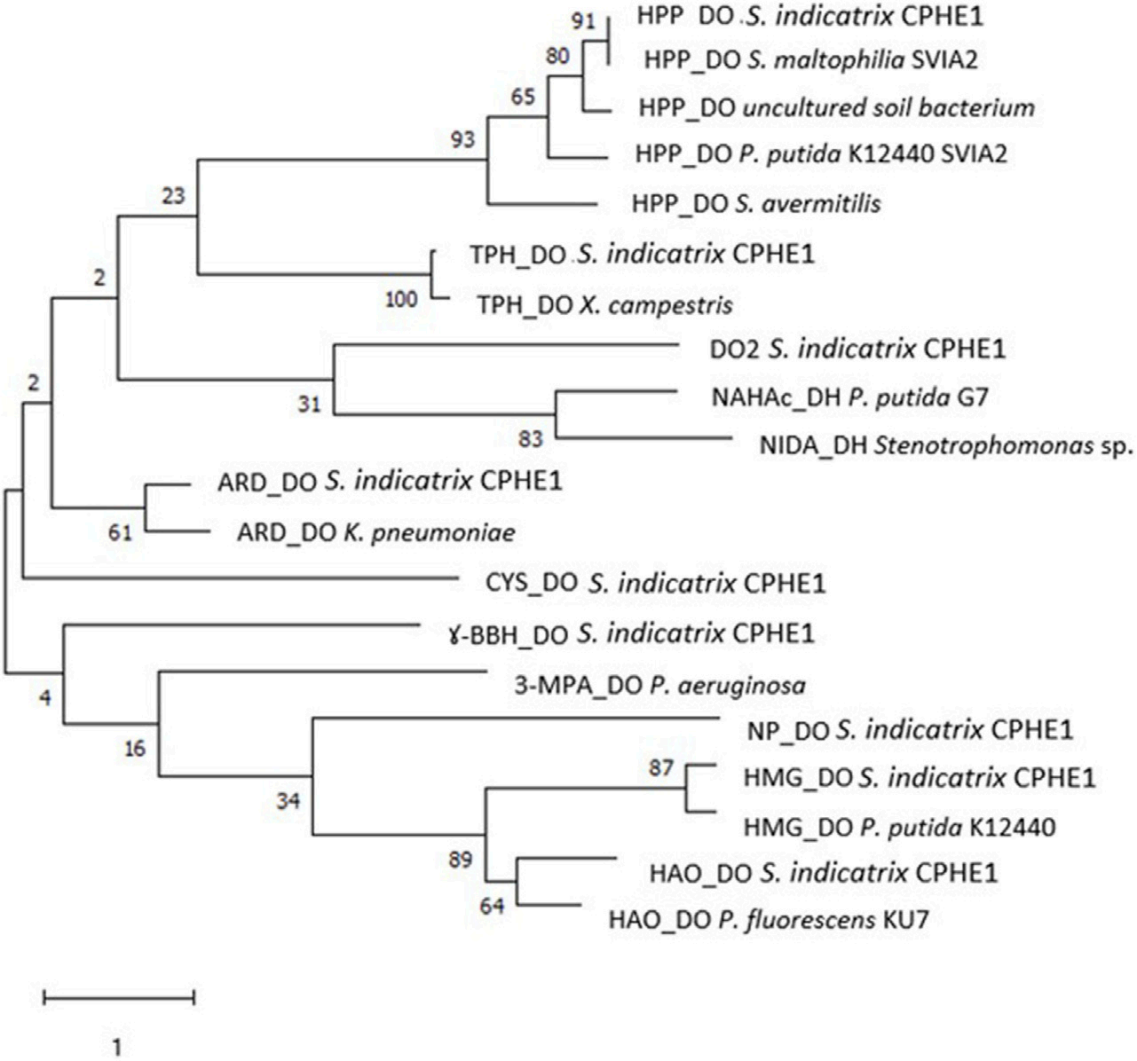
Maximum Likelihood tree based on the dioxygenase protein sequences annotated in the genome of *S. indicatrix* CPHE1 and in the reference dioxygenases involved in the degradation of polycyclic aromatic hydrocarbons. Bootstrap values (>30%) are expressed as percentages of 1000 replicates. Same branches representation was recovered by the neighbour-joining and maximum-parsimony algorithms. The scale bar indicates 1 substitution per nucleotide position in protein sequence tree. Phylogenetic tree was built using MEGA 6.

For this reason, nine dioxygenases annotated in the genome of *S. indicatrix* CPHE1 have been exhaustively analyzed and compared with similar dioxygenases of bacterial genomes deposited in several databases. All dioxygenases have been compared by multiple alignments with reference proteins (Table 2), including two dioxygenases known for their involvement in naphthalene biodegradation (NAHAc of *P. putida* G7 and NIDA of the only naphthalene dioxygenase described in *Stenotrophomonas*) (Cébron et al., 2008; Kumari et al., 2017). As shown in the tree in Figure 2, the enzyme DO2 is the closest phylogenetically to the reference proteins (NAHAc and NIDA). However, annotated dioxygenases have been considered for the subsequent RT-PCR expression experiments since there are no conclusive results to exclude any of them as putative responsible for deoxygenation.

**TABLE 2:** Dioxygenases annotated in *S. indicatrix* CPHE1 genome and reference dioxygenases (literature, pdb, NCBI, etc.).

Name	Function	Strain	References
CYS_DO	Cysteine dioxygenase	*Stenotrophomonas indicatrix* CPHE1	-
3-MPA_DO	3-Mercaptopropionato dioxygenase	*Pseudomonas aeruginosa*	Fellner et al. (2016)
TPH_DO	Tryptophan 2,3-dioxygenase	*Stenotrophomonas indicatrix* CPHE1	-
TPH_DO	Tryptophan 2,3-dioxygenase	*Xanthomonas campestris* TDO	Forouhar et al. (2007)
HPP_DO	4-hydroxyphenylpyruvate dioxygenase	*Stenotrophomonas indicatrix* CPHE1	-
HPP_DO	4-hydroxyphenylpyruvate dioxygenase	*Streptomyces avermitilis*	Brownlee et al. (2004)
HPP_DO	4-hydroxyphenylpyruvate dioxygenase	*uncultured soil bacterium*	Lee et al. (2008)
HPP_DO	4-hydroxyphenylpyruvate dioxygenase	*Stenotrophomonas maltophilia* SVIA2	Elufisan et al. (2019)
HPP_DO	4-hydroxyphenylpyruvate dioxygenase	*Pseudomonas putida* KT2440	Kim et al. (2006)
HAO_DO	3-hydroxyanthranilate 3,4-dioxygenase	*Stenotrophomonas indicatrix* CPHE1	-
HAO_DO	3-hydroxyanthranilate 3,4-dioxygenase	*Pseudomonas fluorescens strain* KU-7	Muraki et al., 2013
HMG_DO	Homogentisate 1,2-dioxygenase	*Stenotrophomonas indicatrix* CPHE1	-
HMG_DO	Homogentisate 1,2-dioxygenase	*Pseudomonas putida* KT2440	kim et al., 2006
DO2	Putative dioxygenase	*Stenotrophomonas indicatrix* CPHE1	-
ARD_DO	1,2-dihydroxy-3-keto-5-methylthiopentene dioxygenase	*Stenotrophomonas indicatrix* CPHE1	-
ARD_DO	1,2-dihydroxy-3-keto-5-methylthiopentene dioxygenase	*Klebsiella pneumoniae*	Dai et al. (2001)
ɣBBH_DO	Gamma-butyrobetaine dioxygenase	*Stenotrophomonas indicatrix* CPHE1	-
NP_DO	2-nitropropane dioxygenase	*Stenotrophomonas indicatrix* CPHE1	-
NP_DO	2-nitropropane monooxygenase	*Pseudomonas aeruginosa* PAO1	Salvi et al. (2014)
NIDA	naphthalene dioxygenase	*Stenotrophomonas* IITR87	Kumari et al. (2017)
NAHAc	naphthalene dioxygenase	*Pseudomonas putida G7*	Cébron et al. (2008)

**FIGURE 3:**
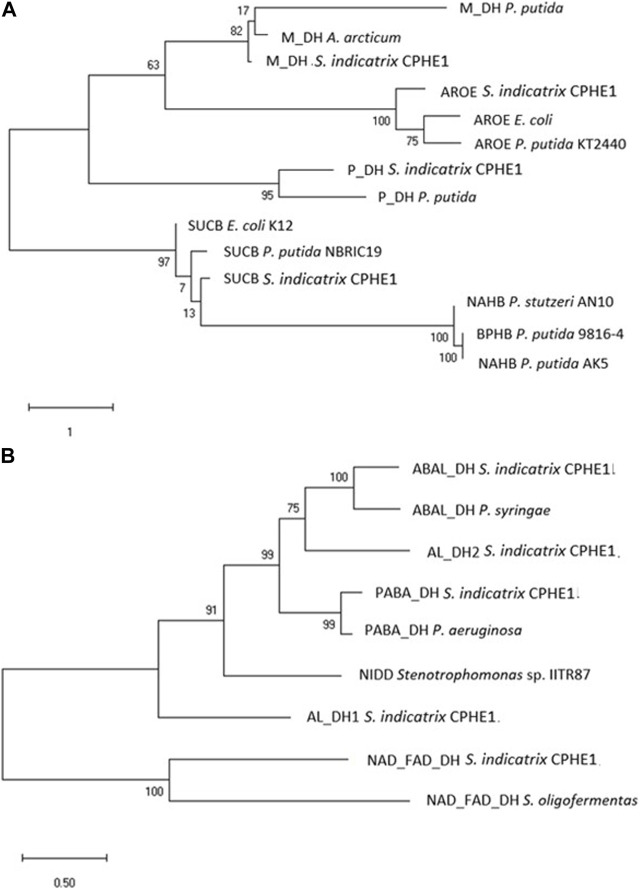
Maximum Likelihood tree based on the dehydrogenase protein sequences annotated in the genome of *S. indicatrix* CPHE1 and reference dehydrogenases NAHB, BPHB, and NIDD **(A)**, and NIDD **(B)** involved in the degradation of polycyclic aromatic hydrocarbons. Bootstrap values (>30%) are expressed as percentages of 1000 replicates. Same branches representation was recovered by the neighbour-joining and maximum-parsimony algorithms. The scale bar indicates 1 or 0.5 substitution per nucleotide position in protein sequence tree. Phylogenetic tree was built using MEGA 6.

After the analysis of the possible dioxygenases, a thorough analysis of the annotated dehydrogenases of *S. indicatrix* CPHE1 was performed, since the dihydrodiol dehydrogenase enzyme catalyzes the second step of the PHE biodegradation route described by Stingley et al. (2004). This enzyme removes two hydrogen atoms from the molecule, oxidizing it, the electrons and protons from the hydrogen atoms are captured by the oxidized form of the coenzyme, which is reduced. Among 119 CPHE1 proteins annotated as dehydrogenases, nine were selected based on their function in relation to the biodegradation of PAHs (Table 3). These amino acid (aa) sequences were analyzed in a phylogenetic tree using diverse dehydrogenase references, including dehydrogenases known as catalysts of the second step of the PAHs biodegradation pathway (NahB, BphB, and NidD). Two different trees were obtained based on the considered aa references (Figure 3); the first tree (Figure 3A) comprises dihydrodiol naphthalene dehydrogenase (NahB) described for *P. stutzeri* AN10, naphthalene dehydrogenase (NahB) of *P. putida* AK5, and cis-2,3-dihydrobiphenyl-2,3-diol dehydrogenase (BphB) of *P. putida* 9816-4. Figure 3B includes naphthalene dehydrogenase (NidD) described in *Stenotrophomonas* sp. IITR87 which resulted in phylogenetically close to five (out of nine) CPHE1 dehydrogenases.

**TABLE 3:** Dehydrogenases annotated in *S. indicatrix* CPHE1 genome and reference dehydrogenases (literature, pdb, NCBI, etc.).

Name	Function	Strain	References
MDH	Malate dehydrogenase	*Stenotrophomonas indicatrix* CPHE1	-
MDH	Malate dehydrogenase	*Pseudomonas putida* ATCC12633	Muramatsu et al. (2004)
MDH	Malate dehydrogenase	*Aquaspirillium arcticum*	Kim et al. (1998)
SUC_B	Dihydrolipoamide succinyltransferase component (E2) of 2-oxoglutarate dehydrogenase complex	*Stenotrophomonas indicatrix* CPHE1	-
SUC_B	Dihydrolipoamide succinyltransferase component (E2) of 2-oxoglutarate dehydrogenase complex	*Pseudomonas putida* NBRIC19	Mishra et al. (2001)
SUC_B	Dihydrolipoamide succinyltransferase component (E2) of 2-oxoglutarate dehydrogenase complex	*Escherichia coli* k12	Spencer et al. (1984)
NAD_FAD_DH	NAD(FAD)-utilizing dehydrogenases	*Stenotrophomonas indicatrix* CPHE1	-
NAD_FAD_DH	NAD(FAD)-utilizing dehydrogenases	*Streptococcus oligofermentas*	Molla et al. (2014)
NAH_B	Cis-dihydrodiol naphthalene dehydrogenase	*Pseudomonas stutzeri* AN10	Bosch et al. (1999)
NAH_B	naphthalene dehydrogenase	*Pseudomonas putida* AK5	Izmalkova et al. (2013)
BPH_B	Cis-2,3-dihydrobiphenyl-2,3-diol dehydrogenase	*Pseudomonas putida* 9816-4	Dennis and Zylstra (2004)
ARO_E	Shikimate 5-dehydrogenase I alpha	*Stenotrophomonas indicatrix* CPHE1	-
ARO_E	Shikimate 5-dehydrogenase I alpha	*Escherichia coli* K12	Michel et al. (2003)
ARO_E	Shikimate 5-dehydrogenase I alpha	*Pseudomonas putida* KT2440	Peek et al. (2014)
ALDH_1	Aldehyde dehydrogenase	*Stenotrophomonas indicatrix* CPHE1	-
NID_D	Naphtalene dehydrogenas	*Stenotrophomonas* sp. IITR87	Kumari et al. (2017)
ALDH_2	Aldehyde dehydrogenase	*Stenotrophomonas indicatrix* CPHE1	-
ABALDH	Gamma-glutamyl-aminobutyraldehyde dehydrogenase	*Stenotrophomonas indicatrix* CPHE1	-
ABALDH	Gamma-glutamyl-aminobutyraldehyde dehydrogenase	*Pseudomonas syringae* DC3000	McClerklin et al. (2018)
PABADH	Betaine aldehyde dehydrogenase	*Stenotrophomonas indicatrix* CPHE1	-
PABADH	Betaine aldehyde dehydrogenase	*Pseudomonas aeruginosa*	Gonzalez-Segura et al. (2009)
PHD	Pyruvate dehydrogenase	*Stenotrophomonas indicatrix CPHE1*	-
PHD	Pyruvate dehydrogenase	*Pseudomonas putida*	Frank et al. (2005)

Although PABA DH (betaine aldehyde dehydrogenase) and SUCB (oxoglutarate dehydrogenase dihydrolipoamide succinyltransferase component) were the closest CPHE1 sequences to the respective reference proteins (for each tree), it should be noted that reference proteins belong to different bacterial genera, consistent in a wide protein sequences variability (Keshavarz-Tohid et al., 2019).

### 3.2 Identification of the involvement of dioxygenases of *S. indicatrix* CPHE1 in phenanthrene degradation by RT-PCR experiments

Studies of gene expression have been carried out by RT-PCR experiments to know which annotated dioxygenase of *S. indicatrix* CPHE1 was expressed throughout the PHE biodegradation process after inoculating CPHE1 strain.

Validation of the samples is reported in Supplementary Figure S2 showing the PCR amplification of 16S rDNA gene using genomic DNA (positive control) from *S. indicatrix* CPHE1, cDNA obtained from RNA extracted from CPHE1 strain without contact with PHE (t_0_) and RNA samples taken at different times from the PHE biodegradation assay (t_1_, t_3_, t_7_, t_14_, and t_21_).

Several authors have used the semi-quantitative RT-PCR to confirm the involvement of genes in the biodegradation pathway of various pollutants. For example, Thanh et al. (2019) observed the expression of *dbfA1A2RBC* genes that share 99%–100% identity with *Paenibacillus* sp. YK5 genes (putatively involved in dibenzofuran degradation). The induction of four of these genes was observed after treatment with dibenzofuran through RT-PCR. Di Canito et al. (2018) studied the role of *Rhodococcus opacus* R7 oxygenases in the *o*-xylene degradation. The important involvement of *akb* genes was demonstrated using RNA obtained from R7 cells in the presence of *o*-xylene by RT-PCR. The result showed that the transcription was induced by the contaminant. In another study, Churchill et al. (2008) checked the ability of *Mycobacterium* sp. CH-2 to mineralize PHE, pyrene, and FLT. Primers were designed based on the dioxygenases *nidAB* and *pdoA2B2* and an alkane monooxygenase. RT-PCR analysis indicated that alkane monooxygenase is constitutively expressed, however, *nidAB*, and *pdoA2B2* were expressed only in the presence of PAHs.

**FIGURE 4:**
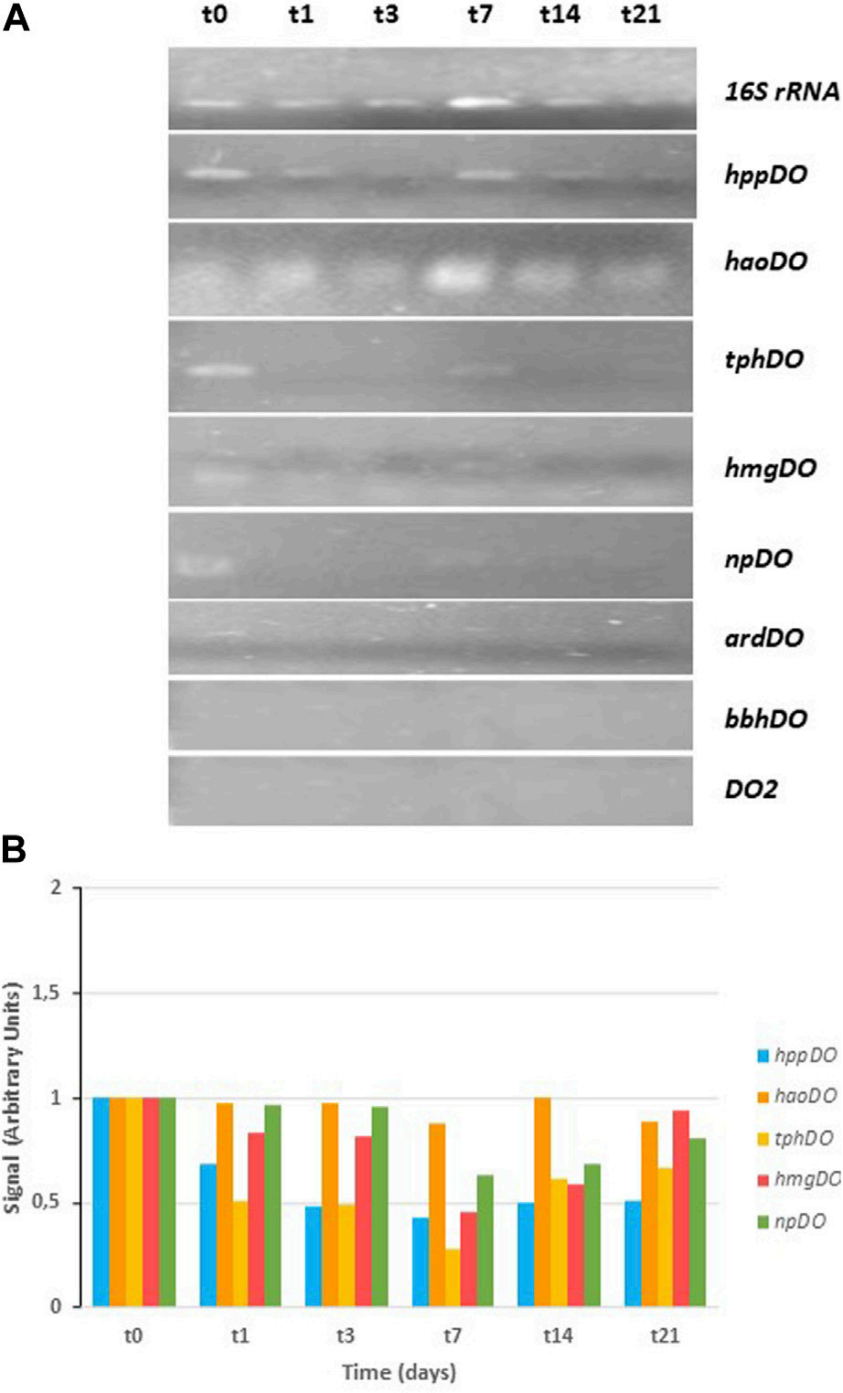
RT-PCR analysis of dioxygenase gene expression in *S. indicatrix* CPHE1. cDNAs were synthesized from RNA at different times (t0, t1, t3, t7, t14, and t21). **(A)** PCR with specific primers for *hppDO*, *haoDO*, *tphDO*, *hmgDO*, *npDO*, *ardDO*, *bbhDO*, and *DO2* (30 cycles) and 16S rRNA (13 cycles) genes were performed. 16S rRNA expression was used as RNA-loading control for these different samples. **(B)** Quantification of dioxygenase expression in relation to the 16S rRNA and negative control (t0) signal is shown in the above histograms (in arbitrary units)..

The results of the CPHE1 dioxygenase gene expression are shown in Figure 4 obtained with specific primers designed on CPHE1 genes reported in Table 2. At the top of the figure are presented the results of the PCR when the 16S rRNA gene was amplified under non-limiting conditions, which serves as a reference gene. Signal intensity quantification (ImageJ.JS) of PCR results indicated that the cDNA of the t_0_, t_1_, t_3_, and t_21_ samples are the least concentrated, followed by the t_14_ sample. The t_7_ sample was twice more concentrated. These data will allow for assessing the expression levels of the rest of the studied genes.

A previous study carried out by Lara-Moreno et al. (2023) showed that the gene annotated as cysteine dioxygenase (*cysDO*) was expressed in the presence of PHE from the third day of treatment. The KEGG database indicates that the *cysDO* enzyme is involved in different biodegradation pathways of aromatic compounds, including naphthalene (M00534), salicylic acid (M00638) and phthalic acid (M00623). In addition, Wang et al. (2017) studied the participation of *cysDO* enzyme in the degradation of aromatic compounds. However, in the currently work, gene expression study has been expanded, all dioxygenases annotated in the genome of CPHE1 strain were analyzed with the aim to know their involvement in PHE biodegradation pathway. The electrophoresis gel obtained is shown in Figure 4. Gene expression of 1,2-dihydroxy-3-keto-5-methylthiopentene dioxygenase (*ardDO*), gamma-butyrobetaine dioxygenase (*bbhDO*) or putative dioxygenase (DO2) was not detected. In the case of tryptophan-2,3-dioxygenase (*tphDO*), homogentisate dioxygenase (*hmgDO*), 4-hydroxyphenylpyruvate dioxygenase (*hppDO*), and nitropropan dioxygenase (*npDO*) genes, a repression of expression was observed in the presence of PHE. And in case of 3-hydroxyanthranilate 3,4-dioxygenase a constituent expression was detected. It concludes that *cysDO* was the only one expressed in the presence of PHE. These results suggest the involvement of a deoxygenation in the PHE degradation pathway when *S. indicatrix* CPHE1 was inoculated. In addition, as indicated above, the presence of dihydrodiol metabolites was detected after inoculating CPHE1 strain (Lara-Moreno et al., 2021). These results suggest the involvement of a dioxygenation in the PHE degradation pathway when *S. indicatrix* CPHE1 was inoculated.

### 3.3 Phenanthrene degrading-genes expression

Beside the dioxygenase gene expression, the expression of other genes involved in PHE biodegradation route was investigated relying on the second bioinformatic approach based on the comparison of *S. indicatrix* CPHE1 genes with sequences belonging to bacterial strains known for their involvement in PHAs biodegradation.

Indeed, Stingley et al. (2004) described the most common PHE biodegradation pathways for *Mycobacterium vanbaalenii* strain PYR-1. These genes were compared to the only two genomic studies (Kumari et al., 2017; Elufisan et al., 2020) that have been published on PHE degradation route in *Stenotrophomonas* genus. For instance, the genes involved in this degradation pathway showed at least 97% identity compared with *Stenotrophomonas* IITR87 genome (Kumari et al., 2017).

Thus, CPHE1 genes showing a high identity with respect to the genes described in the *M. vanbaalenii* PYR-1 genome were taken into consideration as references for the gene expression studies in *S. indicatrix* CPHE1 (Table 4). In addition, genes described in other bacterial species, reported as PAH-degrading, were included in Table 4 as references, accordingly to the hypothesized metabolic pathway.

**TABLE 4:** Reference genes utilized to design the primers for cDNA amplification of *S. indicatrix* CPHE1 at different times.

Gen	% Identity/% positives	References strain	GenBank	Function	References
*nidD*	35/53	*M. vanbaalenii* PYR-1	AAT51749.1	Naphthalene dehydrogenase	Stingley et al. (2004)
*phtB*	35/47	*M. vanbaalenii* PYR-1	AAQ91917.1	Phthalate dihydrodiol dehydrogenase	Stingley et al. (2004)
*pcaL*	37/53	*Rhodococcus hoagii* 103S	CBH49355.1	3-oxoadipate enol-lactone hydrolase/4-carboxymuconolactone decarboxylase	Letek et al. (2010)
*pcaI*	43/58	*Bradyrhizobium diazoefficiens* USDA 110	BAC48727.1	3-oxoadipate CoA-transferase subunit α	Kaneko et al. (2002)
*phtAd*	37/47	*Terrabacter* sp. DBF63	BAC54161.1	Phthalate dioxygenase ferredoxin reductase	Habe et al. (2003)
*catB*	55/72	*Pseudomonas protogens* CHA0	AGL85686.1	Muconate cycloisomerase	-
*phdG*	32/46	*M. vanbaalenii* PYR-1	AAT51745.1	Hydratase aldolase	Stingley et al. (2004)
*benD*	36/50	*P. putida*	BAF02455.1	1,2-dihydroxycIclohexa-3,5-diene carboxylate dehydrogenase	Keil et al. (1985)

**FIGURE 5:**
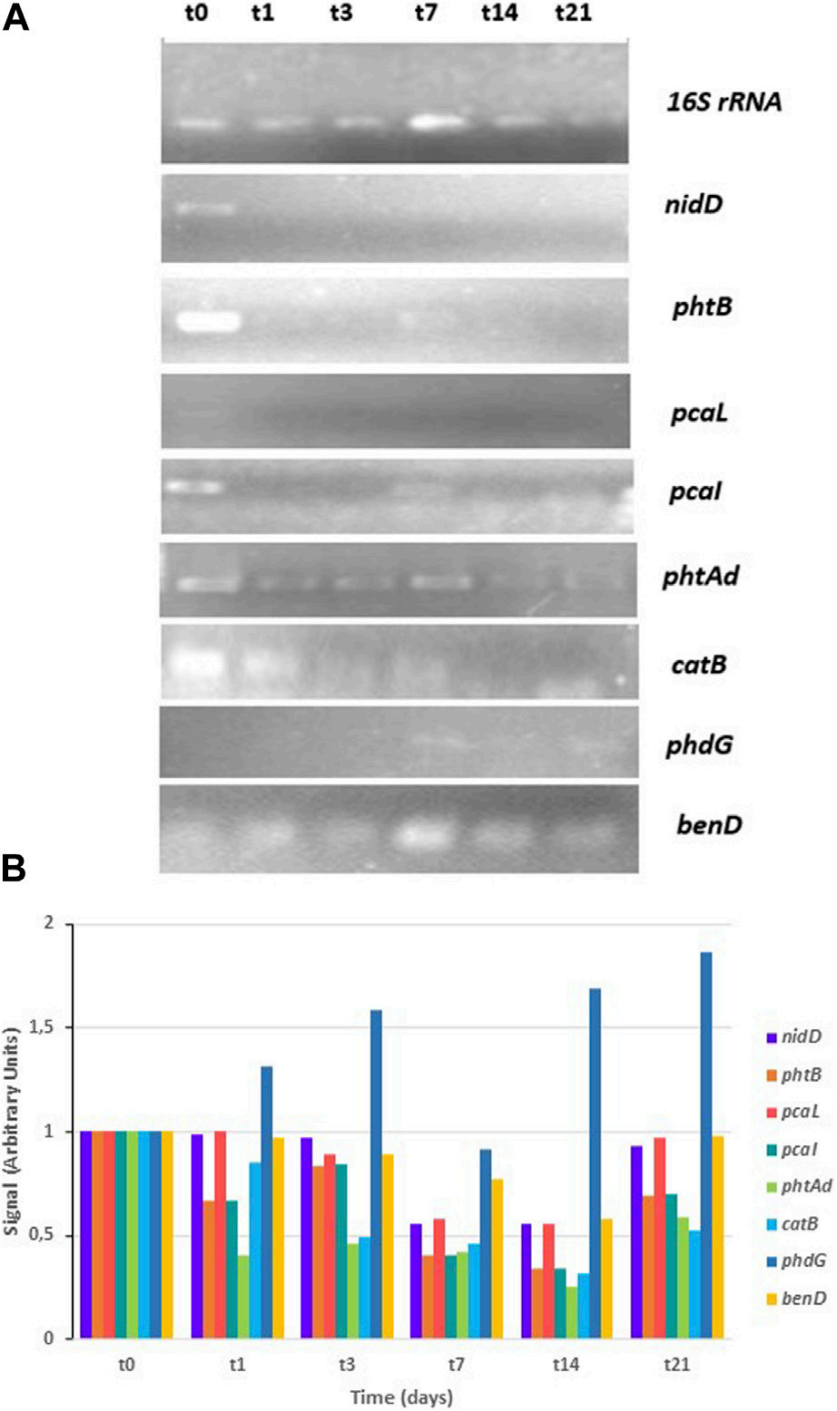
RT-PCR analysis of gene expression in *S. indicatrix* CPHE1. cDNAs were synthesized from RNA at different times (t0, t1, t3, t7, t14, and t21). **(A)** PCR with specific primers for *nidD*, *phtB*, *pcaL*, *pcaI*, *phtAd*, *catB*, and *phdG* (30 cycles) and 16S rRNA (13 cycles) genes were performed. 16S rRNA expression was used as RNA-loading control for these different samples. **(B)** Quantification of studied gene expression in relation to the 16S rRNA and negative control (t0) signal is shown in the above histograms (in arbitrary units).

Results of their expression in the presence of PHE are shown in Figure 5. The expression of the homologous gene encoding aldolase hydratase (*phdG*), responsible for transforming 2-hysroxybenzo(h)chromene-2-carboxylic acid to 4-[1-hydroxy(2-naphthyl)]-2-oxobut-3-enoic acid, was induced after 7 days in contact with the contaminant, suggesting its involvement in the PHE degradation pathway carried out by *S. indicatrix* CPHE1. Usually, in the majority of publications, the gene that encodes the α subunit hydroxylating dioxygenase is identified as a functional marker gene. However, it has been observed that due to low phylogenetic resolution and the lack of specificity, it is derived by erroneous estimation of bacteria that degrade PAHs (Liang et al., 2019). For this reason, Liang et al. (2019) proposed the hydratase-aldolase gene as a biomarker for bacteria involved in PAHs degradation. Therefore, this gene could represent a powerful biomarker to explore the potential of PAH-degrading bacteria in ecosystems, which would have a high impact on the bioremediation of contaminated soils with PAHs. Levieux et al. (2018) and Story et al. (2001) demonstrated that bifunctional enzymes hydratase-aldolase are present in bacterial strains which can use PAHs as the only carbon and energy source, indicating that despite catalyzing an important step in the PAHs degradation, their mechanisms are not very well established.

**FIGURE 6:**
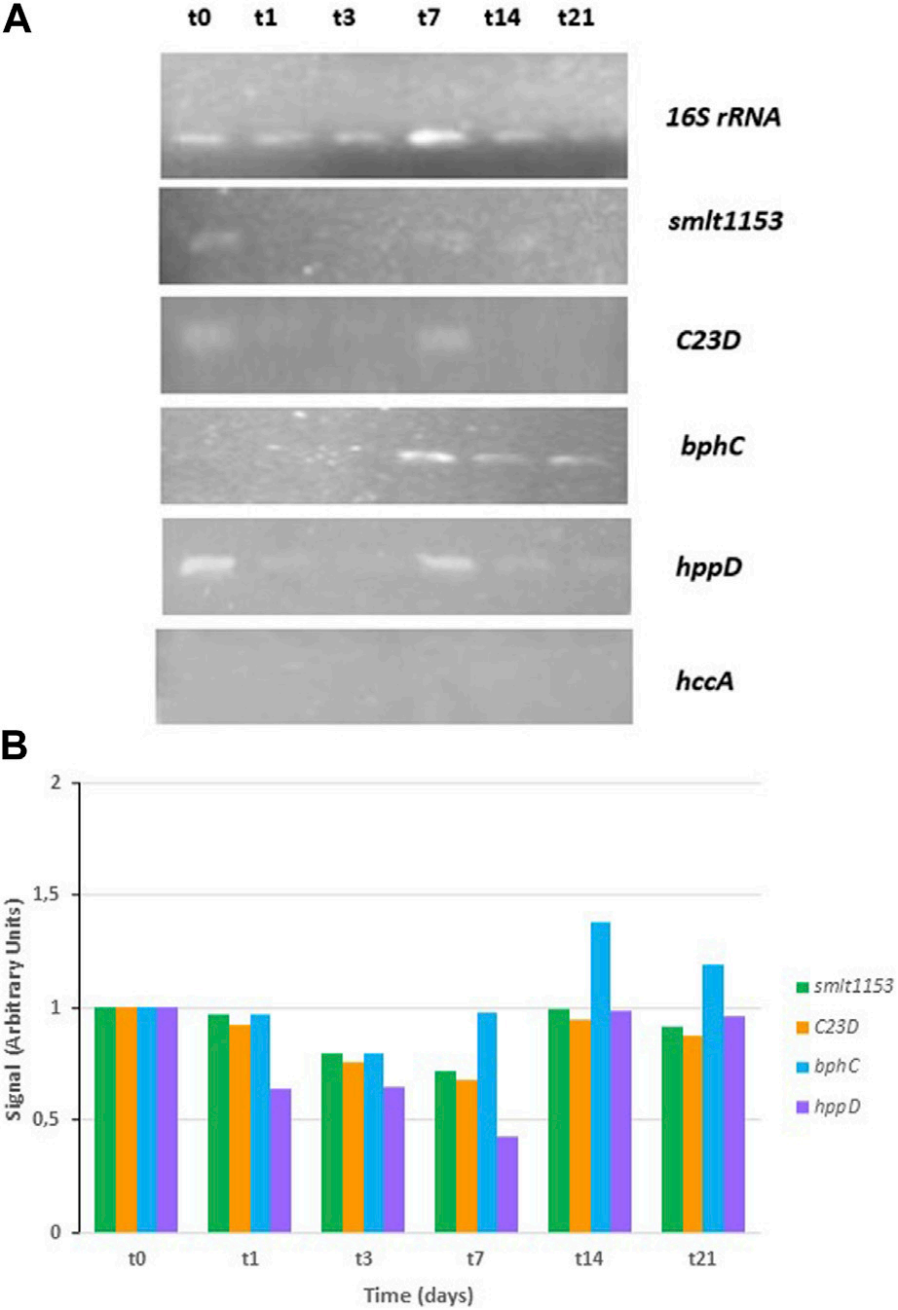
RT-PCR analysis in *S. indicatrix* CPHE1 for the expression study of homologous genes described by Elufisan et al., (2020). cDNAs were synthesized from RNA at different times (t0, t1, t3, t7, t14, and t21). **(A)** PCR with specific primers for *smlt1153*, *C23D*, *bphC* and *hppD* (30 cycles) and 16S rRNA (13 cycles) genes were performed. 16S rRNA expression was used as RNA-loading control for these different samples. **(B)** Studied genes quantification expression in relation to the 16S rRNA and negative control (t0) signal is shown in the above histograms (in arbitrary units).

With the aim of deepening the study of the genes responsible for the degradation of PHE, *Stenotrophomonas* sp. Pemsol genes were also considered, since Pemsol strain can degrade anthraquinone, naphthalene, PHE, phenanthridine, and xylene (Elufisan et al., 2020). Five genes from CPHE1 were selected due to their high homology with Pemsol genes involved in PHAs biodegradation pathway (Table 5). Figure 6 shows the gene expression at different times: the gene encoding catechol-2,3 dioxygenase was constitutively expressed while the expression of biphenyl-2,3-diol 1,2-dioxygenase (*bphC*) was induced after 7 days of treatment, the correspondent gene product catalyzes the transformation of 3,4-hydroxyphenanthrene to 2-acid hydroxycenzochromene-2-carboxylic. *bphC* gene has been also studied in *Sphingomonas*, and *Sphingobium* genera which are able to metabolize PHE via both *meta*- and *ortho*-cleavege pathways (Waigi et al., 2015; Macchi et al., 2019). Moreover, Yun et al. (2014) concluded that PHE induced a strong regulation of *bphC* gene, which is involved in the attack to cleavage the aromatic ring in the lower catabolic route of PAHs in *Novosphingobium pentaromativoransus* species.

**TABLE 5:** *S. indicatrix* CPHE1 genes homologous to the genes of Pemsol strain utilized to design primers for cDNA amplification of *S. indicatrix* CPHE1 at different times.

Gen	% Identity	Function	PEM	References
*smlt1153*	92	Cysteine desulfurase	PEM-03795	Elufisan et al. (2020)
*C23D*	83	Catechol-2,3 dioxygenase	PEM-03383	Elufisan et al. (2020)
*bphC*	92	Biphenyl-2,3-diol 1,2-dioxygenase	PEM-03270	Elufisan et al. (2020)
*hppD*	100	4-hydroxyphenylpyruvate dioxygenase	PEM-03335	Elufisan et al. (2020)

**FIGURE 7:**
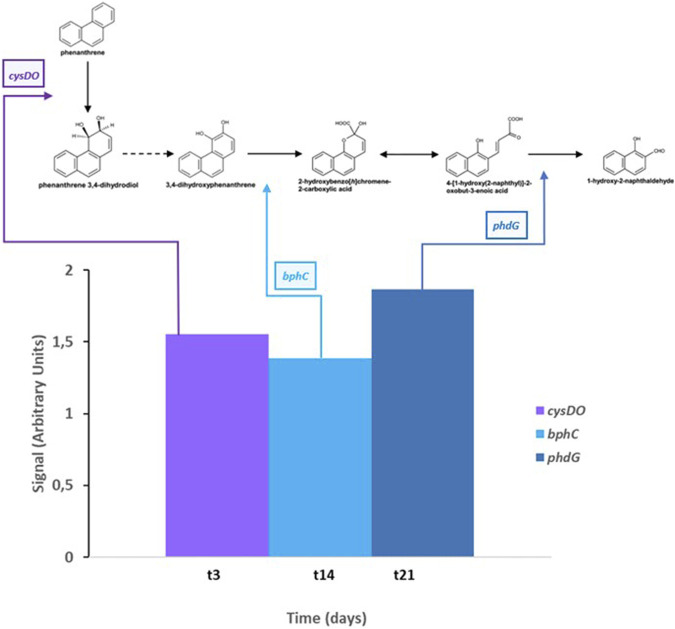
Gene expression results of cysDO (cysteine dioxygenase), bphC (biphenyl-2,3-diol 1,2-dioxygenase), and phdG (aldolase hydratase) according to the time of maximum expression of each gene and step of PHE degradation pathway. Dashed arrow indicates a metabolic step not attributable to a determined gene of S. indicatrix CPHE1; double arrow indicates an isomerase reaction.

All these results together revealed that PHE induces the expression of specific genes (*cysDO*, *phdG*, and *bphC*) in *S. indicatrix* CPHE1 degradation pathway. Figure 7 summarizes the metabolic steps and the time of activation of expressed genes. The gene annotated as *cysDO* was induced after 3 days of inoculation, coinciding with the point where PHE biodegradation begins to be significant (Lara-Moreno et al., 2021), so this dioxygenase could be responsible for catalyzing the first step of the PHE biodegradation pathway. In addition, the induction of *bphC* and *phdG* genes by the presence of PHE from respectively 14 days and 21 days indicates that they could act in intermediate steps as extradiol dioxygenase responsible for the first aromatic ring cleavage and hydratase aldolase responsible for transforming 2-hysroxybenzo(h)chromene-2-carboxylic acid to 4-[1-hydroxy(2-naphthyl)]-2-oxobut-3-enoic acid, respectively.

### 3.4 Phenanthrene mineralization in contaminated soils. Effect of biostimulation, bioaugmentation, and HPBCD application

**FIGURE 8:**
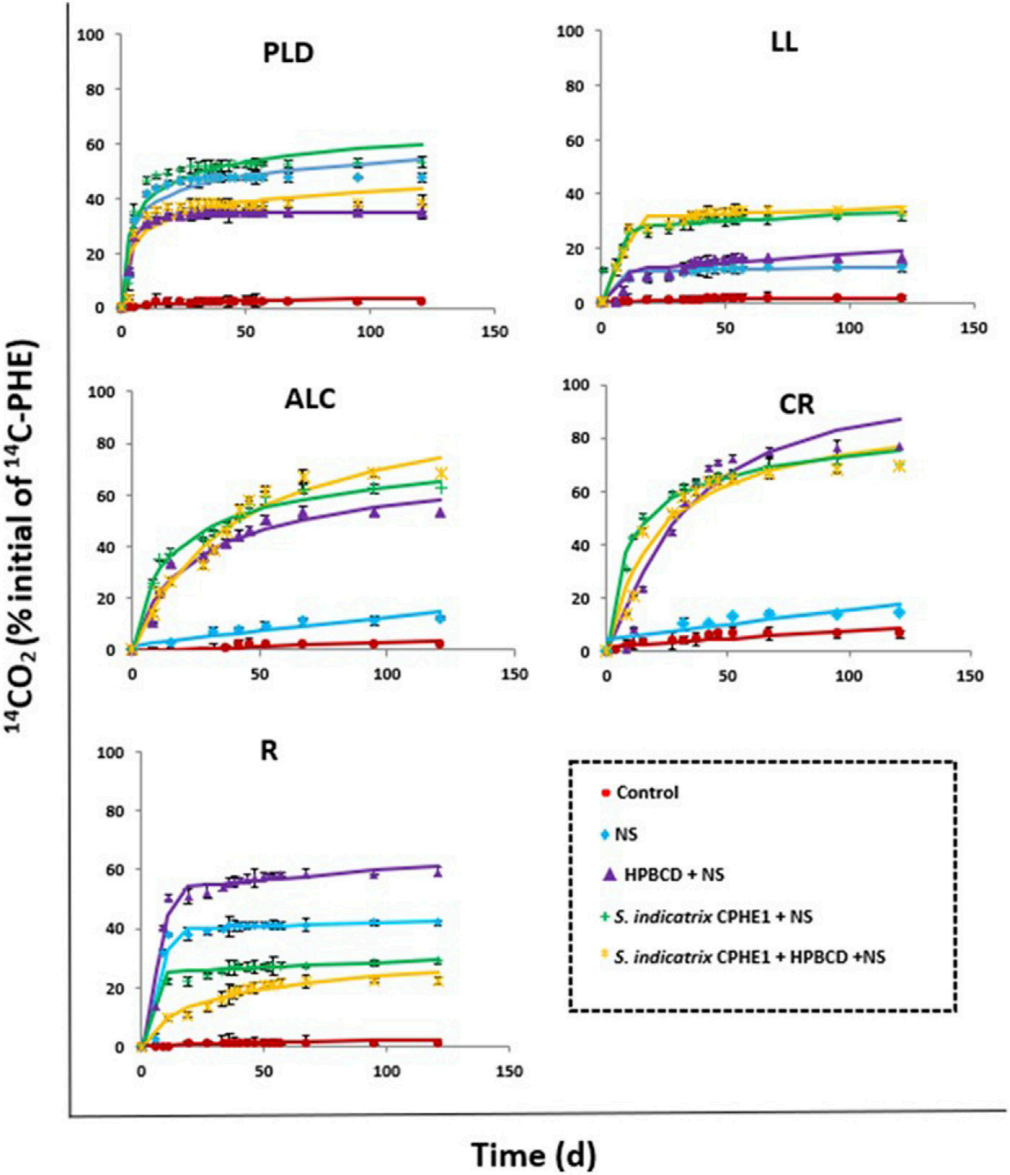
Phenanthrene mineralization (120 d) in PLD, LL, ALC, CR, R soils in slurry systems after application of the different treatments studied.

PHE abiotic dissipation was assessed in the five selected soils, adding HgCl_2_ solution to kill the soil endogenous microbiota. The results concluded that abiotic dissipation was not observed in this study (data not shown). Figure 8 and Table 6 show the PHE mineralization curves and kinetic parameters obtained after the application of the different treatments in the investigated soils. An extension of mineralization of 3.6%, 1.7%, 2.3%, 8.7%, and 1.5% after 120 days for PLD, LL, ALC, CR and R soils was respectively observed when the contaminated soils are not subjected to any bioremediation treatment, with DT_50_ values in the range 1133–6343 days (3–17 years). These results evidenced the need to resort to assisted natural attenuation using biostimulation and bioaugmentation.

**TABLE 6:** Kinetic parameters obtained from phenanthrene mineralization in soils after 120 days.

Soil	Treatment^a^	Kinetic model	K_1_ (d^-1^)	K_2_ (d^-1^)	tb (d)	α (d)	β (d)	DT_50_ (d)	% Extent of mineralization	χ^2^
PLD	A	SFO	2.1 × 10^−4^	-		-	-	3243	3.6	3.1
	B	FOMC	-	-	-	1.4 × 10^−1^	3.6 × 10^−1^	61.1	53.2	6.2
	C	FOMC	-	-	-	6.7 × 10^−2^	7.7 × 10^−2^	2334	38.9	2.6
	D	FOMC	-	-	-	1.7 × 10^−1^	5.9 × 10^−1^	31.5	59.9	7.9
	E	FOMC	-	-	-	1.0 × 10^−1^	3.6 × 10^−1^	361	43.8	5.7
	A	SFO	1.3 × 10^−4^	-		-	-	4213	1.7	0.2
	B	HS	1.8 × 10^−2^	1.7 × 10^−4^	9.6	-	-	3142	13.1	0.3
LL	C	HS	1.4 × 10^−2^	7.2 × 10^−4^	12.3	-	-	732	18.9	0.8
	D	HS	2.7 × 10^−2^	7.3 × 10^−4^	12.2	-	-	499	33.5	0.6
	E	HS	2.4 × 10^−2^	5.1 × 10^−4^	16.1	-	-	614	35.0	0.6
	A	SFO	1.2 × 10^−3^	-	-	-		5643	2.3	1.4
	B	SFO	3.0 × 10^−4^	-	-	-	-	2307	12.3	2.1
ALC	C	FOMC	-	-	-	0.3	8.4	61.6	58.4	4.5
	D	FOMC	-	-	-	0.3	4.9	36.3	65.3	3.9
	E	FOMC	-	-	-	1	40.15	40.5	74.6	6.3
	A	SFO	1.0 × 10^−3^	-	-	-	-	1133	8.7	1.3
	B	SFO	1.2 × 10^−3^	-	-	-	-	573	17.3	1.1
CR	C	FOMC	-	-	-	2.0	64.4	26.4	87.3	12.7
	D	FOMC	-	-	-	0.4	3.5	17.2	75.0	4.9
	E	FOMC	-	-	-	0.7	13.2	25.3	76.9	9.3
	A	SFO	1.3 × 10^−4^	-	-	-	-	6343	1.5	0.2
	B	HS	3.9 × 10^−3^	4.4 × 10^−4^	13.9	-	-	335	42.6	1.3
R	C	HS	5.7 × 10^−2^	1.7 × 10^−3^	14.2	-	-	12.1	61.3	1.6
	D	HS	3.6 × 10^−2^	4.9 × 10^−4^	9.8	-	-	692	29.3	0.3
	E	HS	3.2 × 10^−2^	5.6 × 10^−4^	9.7	-	-	458	24.2	0.8

^a^ Treatments: A) Control (without treatment); B) NS; C) HPBCD + NS; D) *S. indicatrix* CPHE1 + NS; E) *S. indicatrix* CPHE1 + HPBCD + NS.

The addition of inorganic nutrients (NS in Figure 8 and treatment B in Table 6) produced a slight activation of the indigenous microbiota of LL, ALC, and CR soils, and therefore PHE mineralization slightly increased (after 120 days 13.1, 12.3, and 17.3%, respectively). On the contrary, its effect was much greater in the cases of PLD and R soils, in which 53.2% and 42.6% of PHE mineralization were achieved, respectively, decreasing DT_50_ values from 3243 days to only 61.1 d for PLD soil, and from 6343 days to 335 days for R soil. To confirm the presence of PHE-degrading bacterial strains in the studied soils, an enumeration of colony forming units per Gram of soil (CFU g^−1^ soil) in presence of PHE as the only carbon source was carried out (Supplementary Table S3). The CFU counts were in line with the observed mineralization results when the soil microbiota was stimulated using NS. The microbiota of PLD and R soils turn out to be the most active in PHE mineralization, containing the highest number of CFU g^−1^ (1.5 × 10^7^ and 8.6 × 10^7^, respectively) of PHE degrading bacteria; CFU g^−1^ for LL and CR soils were an order of magnitude less than in PLD and R soils, likewise a lower extent of mineralization. ALC soil only reached 12.3% of mineralization and its microbiota was the least rich in potential PHE degraders (1.9 × 10^5^ CFU g^−1^).

OM influences the control of PAH bioavailability in soils (Abdel-Shafy and Mansour, 2016). It has been demonstrated that the interaction between PAHs and the soil increases to longer aging time, causing a decrease in the bioavailability of the pollutants (Ehlers and Loibner, 2006). To increase their bioavailability, extractants such as cyclodextrins have been used. In a previously published work, Morillo et al. (2012) confirmed the formation of inclusion complexes between HPBCD and PHE which caused an increase in its water solubility (376 times higher in the presence of 100 mM HPBCD). HPBCD is considered suitable for application to contaminated soils since the addition of HPBCD to these soils would cause an increase in the concentration of PHE in the soil solution making it more bioavailable (Duán et al., 2021; Lara-Moreno et al., 2022b). Figure 8 (Treatment C) shows an increase in the percentage of mineralization with respect to treatment with only NS (Treatment B) in all cases except in PLD soil. In ALC, CR, and R soils the extent of mineralization reached up to 58.4%, 87.3%, and 61.3%, respectively (Table 6). In addition, the kinetic processes were accelerated, showing a decrease of DT_50_ from 2307, 573 and 335 days to 61.6, 26.4, and 12.1 days after treatment, respectively.

Gao et al. (2014) also evaluated the effect of OM on the biodegradation process and the effect of HPBCD to improve PHE bioavailability. The study concluded that there is a significant inverse relationship between the PHE adsorption capacity to the soil and the extracted fraction with HPBCD and PHE biodegradable fraction. Morillo et al. (2014) performed fluorene (FLU) and fluoranthene (FLT) adsorption-desorption in different soils in the presence of HPBCD. The results confirmed that FLU is more resistant to extraction in soils since its initial adsorption to minerals and OM of the soil is greater due to its smaller size in the case of FLT.

**FIGURE 9:**
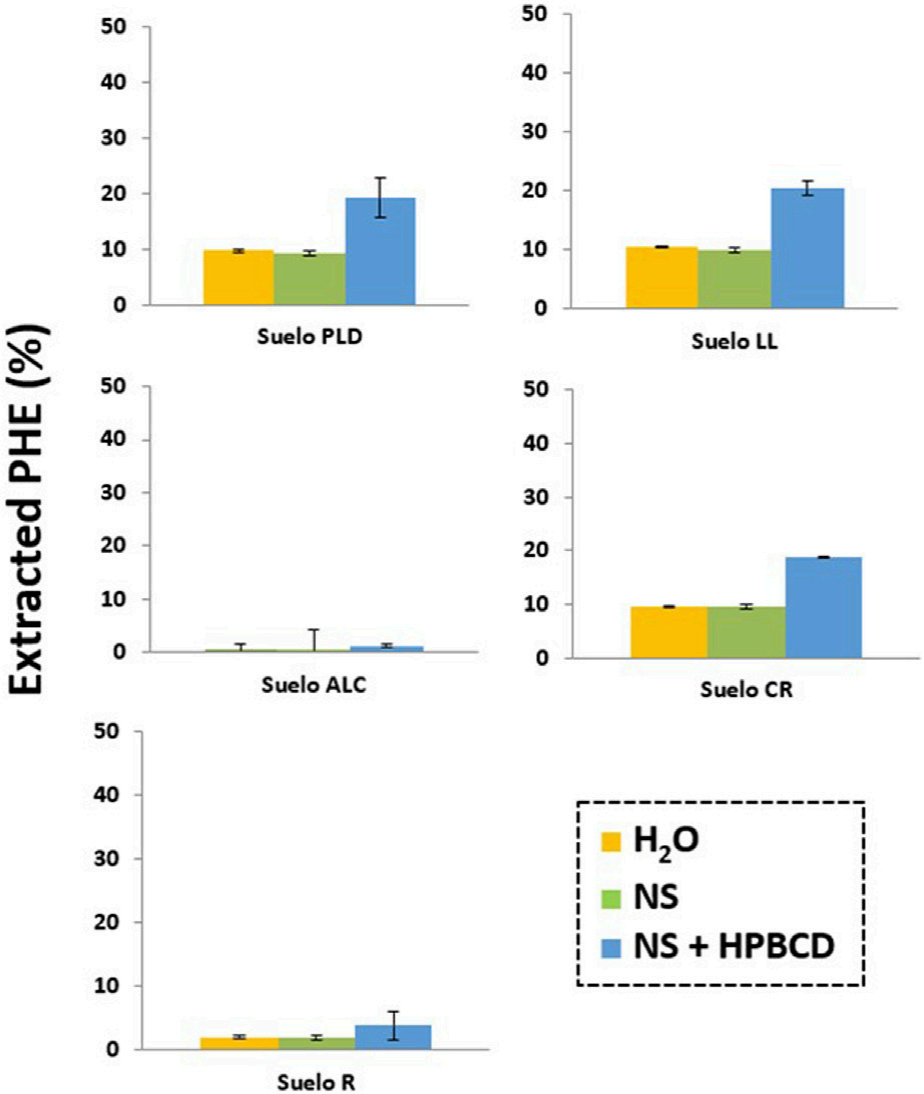
PHE extracted from PLD, LL, ALC, CR, R soil in presence of H20, nutrient solution (NS) and HPBCD.

PHE extraction experiments from soils have been carried out to know the effect produced by NS solution in comparison to HPBCD on the PHE bioavailability (Figure 9). The OM content is very different in the studied soils. This difference influenced the PHE amounts present in the soil solution when NS was added: 9.6% (CR), 10% (LL), 9.2% (PLD), 2% (R), 0.4% (ALC) (listed in increased order of OM content). PHE concentration in the soil solution was higher in all soils when HPBCD was added: 18.8% (CR), 20.4% (LL), 19.2% (PLD), 3.9% (R), 1.2% (ALC), and the observed increase was due to the formation of complexes between HPBCD, and PAHs as previously observed by Morillo et al. (2014). The results confirmed the inverse relationship between the soil OM content (Supplementary Table S1) and the PHE extracted. However, the determined PHE fraction in solution in the presence of NS or HPBCD was not directly correlated with the observed PHE biodegradation curves and the extent of extraction reached for the different soils, concluding that, PHE degradation depends on other factors apart from the fact that HPBCD is able to increase PHE bioavailability.

After a thorough analysis of the CPHE1 strain genome and the degrading genes expression, the ability of *S. indicatrix* CPHE1 to remove PHE was used to improve the bioremediation with a bioaugmentation strategy. When *S. indicatrix* CPHE1 was inoculated in PLD, LL, ALC, and CR soils, improvements in the global extent of PHE mineralization were observed (59.9%, 33.5%, 65.3%, 75%, respectively), and in the mineralization kinetic parameters, reducing the values of DT_50_ (31.5, 499, 36.3, and 17.2 days, respectively). The lowest values of DT_50_ in these 4 soils were obtained when *S. indicatrix* CPHE1 was inoculated as compared to NS or HPBCD application. Shon et al. (2020) observed that *Sphingopyxis soli* KIT-001 showed interactions between the bacterial membrane and PAHs exerting a strong influence on biological processes such as metabolic activity and substrate absorption due to changes in membrane lipids, improving the PHE degradation efficiency.

On the contrary, in the case of contaminated R soil inoculated with *S. indicatrix* CPHE1, a reduction in the extent of mineralization (29.3%) was observed after 120 days, with respect to the degrading capacity shown by the soil microbiota stimulated with NS (42.6%) or the use of HPBCD (61.3%). This fact has been widely observed in the literature because of the competition between the degrading endogenous and exogenous bacteria, which indicates that the presence of *S. Indicatrix* CPHE1 would have partially disabled PHE degradation carried out by the soil endogenous microbiota, and vice versa (Cycoń et al., 2017; Nwankwegu et al., 2022). The presence of a high density of potential PHE-degrading bacteria in R soil (8.6 × 10^7^ CFU g^−1^, Supplementary Table S3) would support this statement.

The combined application of *S. indicatrix* CPHE1 and HPBCD was also tested. The results showed that this co-application clearly increased the extent of PHE mineralization only in soil ALC (with the highest OM content, 13.9%) but the increase was not significant in LL and CR soils, and even a negative effect on the mineralization rate was observed in PLD and R soils in relation to other treatments. Besides, and not less important, DT_50_ values were higher for all soils when *S. indicatrix* CPHE1 and HPBCD were co-applicated, indicating that both treatments should not be added together in these soils.

Previously, Stroud et al. (2009) also observed that HPBCD interfered with the microbial mineralization of PHE and hexadecane resulting in lower mineralization extents, but these authors gave no explanation for this fact. Equally, Villaverde et al. (2019) observed that pyrene mineralization in three soils was not increased or even reduced when using HPBCD together with the exogenous bacterial strains *Achromobacter xylosoxidans* 2BC8 and *S. maltophilia* JR62. The possibility that the increased concentration of PHE in soil solution due to HPBCD addition could be toxic for *S. indicatrix* CPHE1 is discarded because Lara-Moreno et al. (2021) demonstrated its capacity to degrade and mineralize 10 mg L^−1^ PHE in aqueous solution, and its concentration in the soil solution in the present studies is substantially lower. However, since HPBCD could be biodegraded by soil microorganisms (Fenyvesi et al., 2005), the preference of *S. indicatrix* CPHE1 for HPBCD as a carbon and energy source instead of PHE could slow down the mineralization process (Supplementary Table S4).

Each of the studied soils showed different results after the application of the different treatments, so it is considered important to select a bioremediation strategy based on each specific soil and pollutant properties. LL, PLD, ALC, and CR soils required *S. indicatrix* CPHE1 inoculation to improve mineralization kinetics, although in the case of CR soil a higher biodegradation profile was observed only applying HPBCD. The most effective strategy for R soil turned out to be the use of HPBCD, which makes more bioavailable PHE in the soil through the formation of an inclusion complex, since its endogenous microbiota showed to be especially active as PHE-degrader.

## 4 Conclusion

In this work, genome-based analysis and gene expression experiments of *S. indicatrix* CPHE1 involved in PHE degradation unveiled specific genetic determinants that are induced in the presence of PHE. The cysteine dioxygenase (*cysDO*) could be responsible for catalyzing the first step of the PHE biodegradation pathway. *phdG* and *bphC* genes were induced in the presence of PHE later than *cysDO*, in accordance with their role in the degradation pathway, since these enzymes act in intermediate steps, while dioxygenase would act at the beginning of the metabolic pathway. Based on gene expression studies, *S. indicatrix* CPHE1 was selected as a bioaugmentation strategy to bioremediate five soils (PLD, LL, ALC, CR, and R) contaminated with PHE, showing different mineralization results depending on soil properties**.** It is remarkable that *S. indicatrix* has never been described as PHE mineralizing bacterium in soils.

Several authors have isolated PHE-degrading bacteria; however, PAHs contamination remains a major global concern, being biostimulation and bioaugmentation the most useful strategies to conduct decontamination of PAHs polluted environment. In soils with an active microbiota to degrade PHE (soils PLD and R), the only addition of NS was sufficient to achieve PHE mineralization. On the contrary, in the cases of LL, ALC, and CR soils, the inoculation of *S. indicatrix* CPHE1 was crucial to achieving an improvement in PHE mineralization kinetics. HPBCD application provoked an improvement in PHE biodegradation rate in LL, ALC, CR, and R soils, and it was observed that HPBCD was able to achieve better results in the mineralization of PHE in soils with a high OM content. However, the combined application of HPBCD and *S. indicatrix* CPHE1 evidenced a negative effect presumably due to CPHE1 strain preference for CD instead of PHE as a primary carbon source. In general, selecting the best bioremediation strategy based on the physicochemical and microbial properties of each treated soil has been crucial.

## Data Availability

The sequencing data generated were deposited in the National Center for Biotechnology Information (NCBI), under the BioProject ID PRJNA868539. The whole genome shotgun of *S. indicatrix* CPHE1 has been deposited in DDBJ/ENA/GenBank under the accession number JANQDV000000000. The version described in this paper is version JANQDV010000000. The raw data was deposited in the Sequence Read Archive (SRA) under the accession number SRR21098193.
