# Supplementary material for: Genome analysis for the identification of genes involved in phenanthrene biodegradation pathway in Stenotrophomonas indicatrix CPHE1. Phenanthrene mineralization in soils assisted by integrated approaches
Source: Front Bioeng Biotechnol. 2023 May 4;11:1158177. doi: 10.3389/fbioe.2023.1158177 (PMC10192627; doi:10.3389/fbioe.2023.1158177)
Supplement: Supplementary file 1 [file Table1.DOCX]

**Table 1S.** Some properties of the soils used.

| **Soils** | **pH** | **CO_3_^-2^ (%)** | **OM (%)** | **Sand (%)** | **Silt (%)** | **Clay (%)** | **Textural classification** |
| --- | --- | --- | --- | --- | --- | --- | --- |
| **PLD** | 8.24 | 9.70 | 1.67 | 47.0 | 17.1 | 34.5 | Clay loam |
| **LL** | 7.84 | 4.00 | 0.87 | 79.6 | 9.30 | 11.1 | Sandy |
| **ALC** | 5.1 | 0.5 | 13.9 | 69.1 | 7.80 | 23.1 | Sandy loam |
| **CR** | 8.68 | 11.6 | 0.66 | 73.9 | 16.1 | 10.0 | Sandy |
| **R** | 7.72 | 4.00 | 3.44 | 77 | 9.50 | 13.5 | Sandy |
|  |  |  |  |  |  |  |  |
