# Supplementary figures and images for: Genome analysis for the identification of genes involved in phenanthrene biodegradation pathway in Stenotrophomonas indicatrix CPHE1. Phenanthrene mineralization in soils assisted by integrated approaches
Source: Front Bioeng Biotechnol. 2023 May 4;11:1158177. doi: 10.3389/fbioe.2023.1158177 (PMC10192627; doi:10.3389/fbioe.2023.1158177)

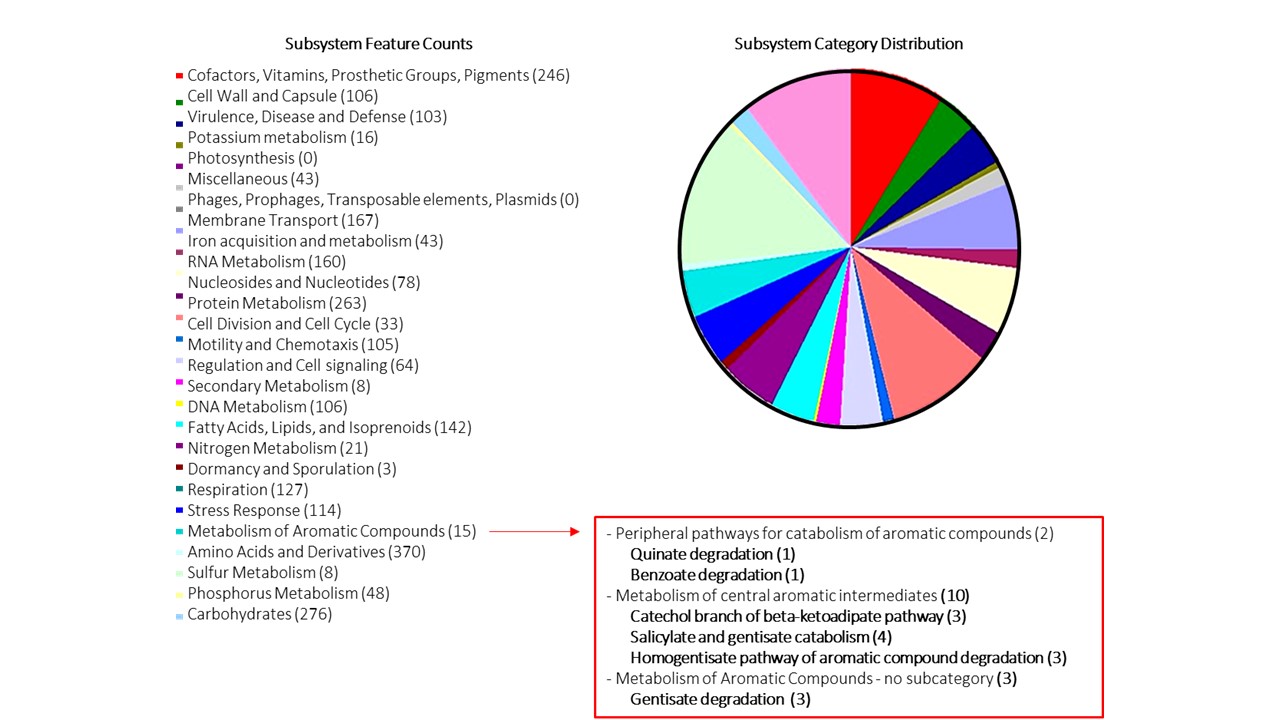

Supplement: Supplementary file 2 [file Image1.JPEG]

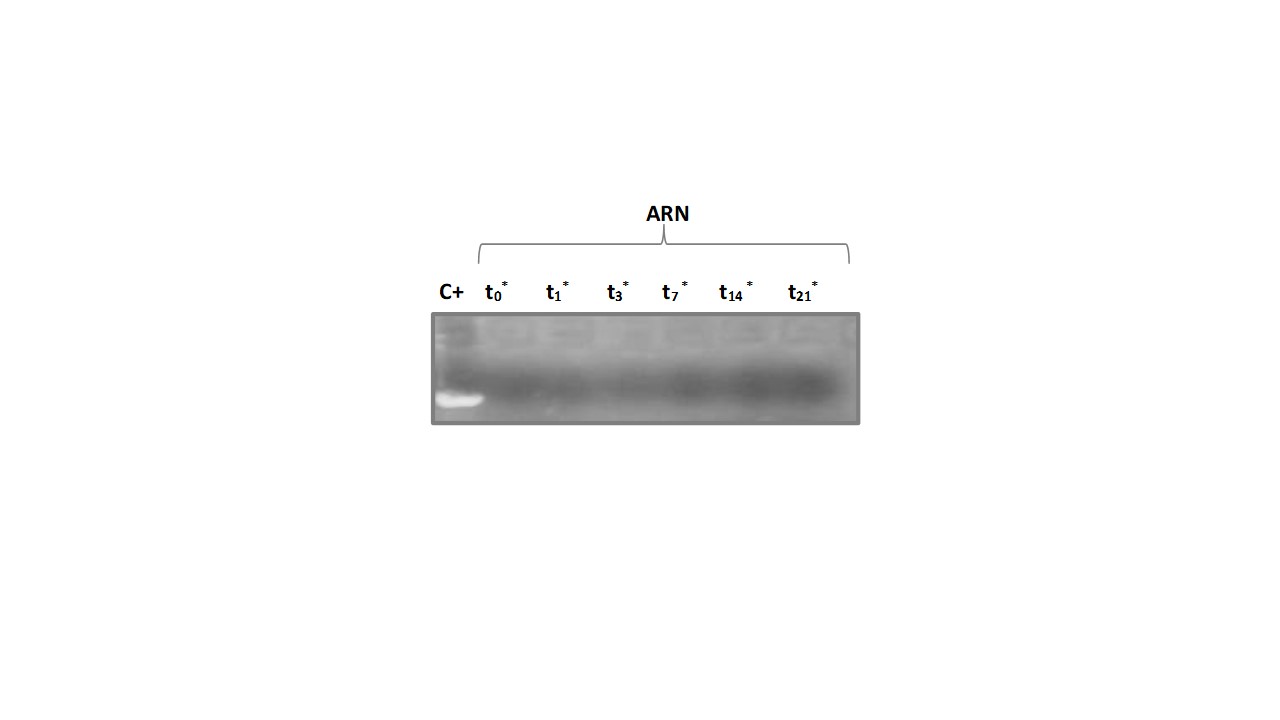

Supplement: Supplementary file 3 [file Image2.JPEG]
