# Supplementary material for: Genome analysis for the identification of genes involved in phenanthrene biodegradation pathway in Stenotrophomonas indicatrix CPHE1. Phenanthrene mineralization in soils assisted by integrated approaches
Source: Front Bioeng Biotechnol. 2023 May 4;11:1158177. doi: 10.3389/fbioe.2023.1158177 (PMC10192627; doi:10.3389/fbioe.2023.1158177)
Supplement: Supplementary file 4 [file Table2.DOCX]

| **Primers ^(1)^** | **Secuence (5’🡪3’)** | |
| --- | --- | --- |
| **16F27 ^(2)^** | AGAGTTTGATCCTGGCTCAG | |
| **16R1488 ^(2)^** | | CGGTTACCTTGTTAGGACTTCACC |
| ***phdG*_F** | | GATCTCAGTCGGTTCCAACG |
| ***phdG*_R** | | GACACCGCAGAAGTCATGG |
| ***pcaL*_F** | | GTGATCCACGGTGATGATGA |
| ***pcaL*_R** | | GGTCCTGATTGACCTTGTCC |
| ***phtAd*_F** | | ATCTGGAAGCTCGCTCACAT |
| ***phtAd*_R** | | GGCATTGCTTGCTTTTTCAC |
| ***nidD*_F** | | CATCTGCTGGATCAACACCT |
| ***nidD*_R** | | GCGTAGTCACCCAGTTCGAC |
| ***phtB_F*** | | TCGACATCCTGGTCAACAAC |
| ***phtB*_R** | | CACCCTTGGTAGCCGAGTAA |
| ***benD*_F** | | CAGATGACCCGCAACCTC |
| ***benD*_R** | | CGTCCGATCACTTCCTCGTA |
| ***catB*_F** | | GGTAGCCAAGAGCGATGTGA |
| ***catB*_R** | | GATCTCCGACTCGTTGAAGC |
| ***pcaI*_F** | | TCGTTGAAGTGGAGGAGGTC |
| ***pcaI*_R** | | GCGCTGTTCGATACGTTTTT |
| ***cysD*_F** | | AGCTGATCGTGACCCCGTA |
| ***cysD*_R** | | GCTCCATTACGCCTTGGTAG |
| ***ardD*_F** | | AGATCGAGTGCGTGAAGGAT |
| ***ardD*_R** | | GGAATGTAACGGGGGAATTT |
| **Primers ^(1)^** | | **Secuence (5’🡪3’)** |
| ***tphD*_F** | | AACGCATCTACCAGGACACC |
| ***tphD*_R** | | AGGGAAGAAGGTCAGTTCCAG |
| ***bbhD*_F** | | GAAATCCTGGTGCTCGACAA |
| ***bbhD*_R** | | ACCTTGAAGCCCAGTTCCAG |
| ***hmgD*_F** | | GTCGAAGGCCTGTACACGAT |
| ***hmgD*_R** | | GTCGGCGTCGTAGAAGTAGC |
| ***hppD*_F** | | CAACGAGTACCTGGATGCGT |
| ***hppD_*R** | | TCGGCGTCGATCAGAATCTT |
| ***npD*_F** | | GGTGATGCCCGATGAAGTC |
| ***npD*_R** | | GATCGTAACTGCCGAACCAC |
| ***hao*_F** | | AAAACCGGCATCTGTTGAAG |
| ***hao*_R** | | GCCTTCGTCGTAGTGGAAAT |
| ***DO2*_F** | | GTGCAGGTCGGTTTTACGAT |
| ***DO2*_R** | | TCAGGCACAACAGGTCTTCA |
| ***C23D_*F** | | GCCTCGCTGGACTTCTACAC |
| ***C23D_*R** | | ACGGAGCCTTCTTGGTTCTC |
| ***Smlt1153_*F** | | GCGTGGAGATGAACCGTG |
| ***Smlt1153_*R** | | CTTCCGGCTTGGGAATGGT |
| **bphC_F** | | GGCAAGACCACCTGCTTCTA |
| **bphC_R** | | CTGTTCGGAAACATGCCCCA |
| ***hcca_*F** | | TTCGTCTCGCCCTATTCCTA |
| ***hcca_*R** | | AGATCCAGTCGAACAGCACA |
| ***hppd_*F** | | CAACCTGTACTTCGGCAACA |

1. *The primers indicated with F is referred to Forward sense and R to Reverse sense.*
2. *Lane, 1991*
