# Supplementary material for: Genome analysis for the identification of genes involved in phenanthrene biodegradation pathway in Stenotrophomonas indicatrix CPHE1. Phenanthrene mineralization in soils assisted by integrated approaches
Source: Front Bioeng Biotechnol. 2023 May 4;11:1158177. doi: 10.3389/fbioe.2023.1158177 (PMC10192627; doi:10.3389/fbioe.2023.1158177)
Supplement: Supplementary file 5 [file DataSheet1.PDF]

**CAPTIONS**

**Figure 1S.** Graphical distribution of the different metabolic subsystems identified in the genome of *S. indicatrix* CPHE1.

**Figure 2S.** 16S rRNA PCR products in agarose gel electrophoresis (2% w/v) using DNA (C+) and RNA samples extracted from *S. indicatrix* CPHE1 at different times of PHE biodegradation. * Study time expressed in days.
