# Supplementary material for: Genome analysis for the identification of genes involved in phenanthrene biodegradation pathway in Stenotrophomonas indicatrix CPHE1. Phenanthrene mineralization in soils assisted by integrated approaches
Source: Front Bioeng Biotechnol. 2023 May 4;11:1158177. doi: 10.3389/fbioe.2023.1158177 (PMC10192627; doi:10.3389/fbioe.2023.1158177)
Supplement: Supplementary file 6 [file Table3.DOCX]

| **Soil** | **CFU g^-1^** |
| --- | --- |
| PLD | 1.5x10^7^ |
| LL | 1.5x10^6^ |
| ALC | 1.9x10^5^ |
| CR | 4.0x10^6^ |
| R | 8.6x10^7^ |

**Table 3S.** Determination of specific PHE-degrading microorganisms in the studied soils.
