# Supplementary material for: Genome analysis for the identification of genes involved in phenanthrene biodegradation pathway in Stenotrophomonas indicatrix CPHE1. Phenanthrene mineralization in soils assisted by integrated approaches
Source: Front Bioeng Biotechnol. 2023 May 4;11:1158177. doi: 10.3389/fbioe.2023.1158177 (PMC10192627; doi:10.3389/fbioe.2023.1158177)
Supplement: Supplementary file 7 [file Table4.DOCX]

**Table 4S.** Bacterial growth under different conditions.

| **Bacterium** | **LB** | **LB 1:40** | **LB 1:40 + PHE** | **LB 1:40 + HPBCD X10** | **LB 1:40 + HPBCD X10 + PHE** |
| --- | --- | --- | --- | --- | --- |
| *S. indicatrix* CPHE1 | +++++ | + | ++ | +++ | ++++ |

**+:** Level of growth: (+++++) maximum growth, (+) minimal growth.
**LB 1:40:** Diluted 1:40 LB broth.

**HPBCD X10:** 10 times the molar concentration of PHE previously added in soil.
